# Analysis of Chromosome 17 miRNAs and Their Importance in Medulloblastomas

**DOI:** 10.1155/2015/717509

**Published:** 2015-03-19

**Authors:** Sebastian López-Ochoa, Marina Ramírez-García, Eduardo Castro-Sierra, Francisco Arenas-Huertero

**Affiliations:** ^1^Laboratory of Research in Experimental Pathology, Hospital Infantil de Mexico Federico Gomez, Avenida Dr. Márquez 162, Colonia Doctores 06720, México, DF, Mexico; ^2^Laboratory of Psicoacoustics and Auditivy Physiology, Hospital Infantil de Mexico Federico Gomez, Avenida Dr. Márquez 162, Colonia Doctores 06720, México, DF, Mexico

## Abstract

MicroRNAs (miRNAs) are small sequences of nucleotides that regulate posttranscriptionally gene expression. In recent years they have been recognized as very important general regulators of proliferation, differentiation, adhesion, cell death, and others. In some cases, the characteristic presence of miRNAs reflects some of the cellular pathways that may be altered. Particularly medulloblastomas (MB) represent entities that undergo almost characteristic alterations of chromosome 17: from loss of discrete fragments and isochromosomes formation to complete loss of one of them. An analysis of the major loci on this chromosome revealed that it contains at least 19 genes encoding miRNAs which may regulate the development and differentiation of the brain and cerebellum. miRNAs are regulators of real complex networks; they can regulate from 100 to over 300 messengers of various proteins. In this review some miRNAs are considered to be important in MB studies. Some of them are miRNA-5047, miRNA-1253, miRNA-2909, and miRNA-634. Everyone can significantly affect the development, growth, and cell invasion of MB, and they have not been explored in this tumor. In this review, we propose some miRNAs that can affect some genes in MB, and hence the importance of its study.

## 1. Medulloblastoma

Medulloblastoma (MB) is the embryonal tumor of the cerebellum and the most common intracranial embryonal tumor. It develops in the posterior fossa, where there are subconscious motor nuclei of great importance, such as those of balance, posture, speech, swallowing, and other important functions.

It is the tumor with the second highest incidence in childhood and constitutes 20% of all childhood central nervous system (CNS) tumors, 70% of which occur in patients under 16 years. Its peak incidence is at age 7, but there are also reports of prenatal and neonatal cases [1, 2]. Seventy-five percent of these tumors are located in the vermis and show very characteristic features in neuroimaging, which allows identifying them [3]. Despite their intratumoral heterogeneity, the variety of histological subtypes, and the irregularity and variety of immunohistochemical results for different proteins, the differential diagnosis can be relatively simple with adequate clinical and radiological information, even in the case of an intraoperative frozen biopsy or a partial resection. High cell density, abundant mitosis, and apoptosis, as well as a great tendency to subarachnoid infiltration, are common features in all variants of MB [2, 3]. The identification of these subtypes has sometimes prognostic implications or can involve pathogenically separate groups. The World Health Organization [4] currently classifies MB in the following:

1. classic MB;
2. desmoplastic/nodular MB;
3. MB with extensive nodularity;
4. large-cell MB;
5. anaplastic MB.

## 2. MB Subtypes

Before the 90s, MB was considered as a histologically uniform entity. In the year 1992, Giangaspero et al. identified a large-cell MB that also corresponded to a more aggressive MB group [5]. The cells in these tumors show large vesicular nuclei with prominent nucleoli and also frequently show amplification of the oncogene c-myc and an isochromosome 17q. This subtype of MB is associated with a poor prognosis; it spreads easily through the cerebrospinal canal. In 2000, Brown et al. analyzed a large group of 495 MB and found a large number of cases with large/anaplastic nuclei that also had different histologic and cytogenetic features [6]. Later, Lamont et al. (2004) demonstrated the usefulness of combining histopathologic features and molecular alterations to stratify patients with MB [7]. This group of patients with anaplastic MB and loss of chromosome 17 has lower survival rates than patients without loss of this chromosome. Afterwards, it was shown that chromosome 17 and its alterations are important markers to stratify patients with respect to their prognosis [8].

Finally, broader and deeper studies of MB using gene expression profiles, gene-microarray analysis, and gene polymorphism analysis, among other methods, have led specialists to establish a consensus that MB can be classified into the following 4 subgroups [9]:

 subgroup 1, wingless-type (WNT);
 subgroup 2, sonic hedgehog (SHH);
 subgroup 3;
 subgroup 4.

Each of these MB subgroups has characteristic molecular profiles and genetic alterations.

### 2.1. WNT Subgroup

To this subgroup belong between 10 and 15% of all MB cases [10]. This subgroup is characterized because 90% of the cases belonging to it present the typical histology of an MB; the patients are older than 3 years old (it can also occur in adults but never in children under 3 years); it has a good prognosis and rarely shows metastasis [11, 12].

### 2.2. SHH Subgroup

This subgroup comprises 25–30% of all MB cases. It is characterized by the presence of a desmoplastic reaction in histopathological analysis (40%). It occurs in patients under 3 years old or in very young adults over 16 years [13]. Half the adult cases of MB also belong to this subgroup. The prognosis of MB in this subgroup is good in patients under 3 years old and very young adults [14].

### 2.3. Subgroups 3 and 4

These subgroups were originally designated as non-WNT/non-SHH. The cases have some similarities in clinical presentation and molecular profile. Most of them have a typical histological pattern; a desmoplastic reaction may occur but is rare. Most cases are in children in both age groups. Both subgroups show a similar frequency of metastasis, but the cases in subgroup 3 have a poor prognosis while the cases in subgroup 4 have an intermediate prognosis [10, 14, 15].

Several cytogenetic and molecular biological studies have confirmed that the etiology of MB is related to the deletion of the short arm of chromosome 17 (17p) and that this deletion occurs in 25–50% of cases. Thus, it is important to study the genetic and molecular mechanisms regulating noncoding RNAs that form complex networks, such as in microRNAs (miRNAs), with at least 100 different genes. Thus, using a different approach to study and analyze MB cases has interesting and important consequences, as will be described later [16].

## 3. Importance of Chromosome 17

Chromosome 17 is one of the 23 pairs of human chromosomes; the anomalies and functions that have been studied with respect to the expression of the genes of this chromosome affect, among other organs, the nervous system, particularly the differentiation and cell and tissue maturation process.

In addition, several articles and databases that align the sequence of miRNAs and estimate mRNA targets suggest that much of the miRNAs encoded on chromosome 17 have regulatory activity at different stages of neuronal differentiation.

Mutations in the tumor protein 53 gene (TP53) and deletions in the 17p region are among the most common disorders encountered in primary solid tumors of different histological origin [17]; both types of disorders induce genomic instability in transformed cells. It has also been observed that the inactivation or loss of the TP53 gene is associated with a poor prognosis and advanced stages of cancer and with the progression of various neoplasias [18], suggesting that the inactivation of this gene or the deletion of the 17p region is of great importance in carcinogenesis, as observed in other examples such as breast, stomach, liver, and colon cancer and in head tumors [18, 19].

## 4. Chromosome 17 in MB

The first reports on the importance of the alterations of chromosome 17 in MB come from the work of Emadian et al. [20] and Steichen-Gersdorf et al. [21]. These studies showed that the allelic loss of regions of chromosome 17 is associated with poor prognosis when compared with the prognosis of patients with MB without these alterations.

The analysis of chromosome 17 alterations in four MB tumor lines and in an induction model of tumor implant showed that there are different types of alterations including the presence of a dicentric chromosome i(17q), two normal copies of chromosome 17, loss of telomere in 17p, and deletion in 17p11.2 [22].

Other studies have shown that loss of 17p and the gain of 1q correlated with poor survival. The gain of 17q without loss of 17p showed a tendency to better prognosis. The careful analysis of all data suggested that, in general, the loss of 17p is a marker of poor prognosis, while the gain of 17q might be a new marker of good prognosis in patients with MB [8].

Based on these results, it can be noted that good and poor prognosis groups cannot be accurately differentiated according to the alterations of chromosome 17, but what is clear is the presence of genes that are important for the development of CNS, and that despite the great clinical utility of classifying the different subgroups of MB, these markers do not seem to discern in more detail the patients within these subgroups from the point of view of prognosis, strongly justifying the proposal of slightly finer markers able to discriminate between subgroups of response to treatment and that can reflect or participate in important or critical pathways of MB, especially in pediatric cases. Thus, some miRNAs are proposed as new markers, in this case molecular markers in MB that can be used to subclassify and distinguish between groups of good and poor prognosis.

## 5. Molecular Markers

A marker is a character or a gene that, due to linkage, may be used to indicate the presence of another gene; molecular markers are thus a necessary tool in many fields of biology such as the study of evolution, ecology, biomedicine, forensics, and diversity studies. In addition, they are used to locate and isolate genes of interest. At present, there are several molecular techniques that allow us to know indirectly what the proportions of genes in natural populations are as with the analysis of proteins or, in a direct manner, with DNA studies [23].

Advances in the study of molecular pathways, the identification of biomarkers, and new therapies have been important for the development of new methods of characterization and clinical management; they have also expanded the understanding of the molecular pathogenesis of some types of cancer [24].

In the case of MB, important advances have also been made in the study of the major gene-regulated pathways that are altered in this tumor. In fact, some of these pathways are related to signaling pathways during cerebellar development in the embryonic stage. Some of these pathways are the SHH, WNT, and that of the gene associated with notches in the edges of the wings in* Drosophila* (NOTCH). The deregulation of these pathways strongly affects cerebellar development and may participate in the formation of MB [16].

### 5.1. SHH Pathway

The sustained expression of the SHH pathway causes serious cerebellar disorders such as development of MB [25].

The association between the overactivation of the SHH pathway and the development of MB arose from the finding that patients with Gorlin syndrome are predisposed to the development of multiple tumors, including MB [26].

Later the genetic analysis studies came that showed that the patched gene (PTCH) gene of the SHH pathway is mutated in these patients [27, 28].

Mutations of other genes in this pathway have been subsequently described, such as smoothened (SMO) in 5% [29] and suppressor of fused (SUFU) in 9% of patients [30].

### 5.2. WNT Pathway

The first evidence demonstrating the involvement of the WNT pathway in the development of MB came from the genetic study of patients with Turcot syndrome, who are 92 times more likely to develop MB than the general population. These patients carry a germline mutation of the adenomatous polyposis coli gene (APC) gene involved in the WNT pathway [31]. Subsequently, it was shown that other MB cases also had mutations in other genes of the WNT pathway such as *β*-catenin and axin 1 [32, 33].

### 5.3. NOTCH Pathway

The Notch protein is a transmembrane protein and exists as a heterodimeric receptor. The extracellular domain contains repeated domains similar to the epidermal growth factor and is involved in the binding of ligands, preventing signaling in the absence of these. The participation of NOTCH-1 and NOTCH-2 in MB has been described. NOTCH-1 inhibits the proliferation of MB while NOTCH-2 promotes cell growth [34]. Furthermore, the expression of NOTCH-1 is so low that it is undetectable in some cases, while NOTCH-2 is overexpressed in MB [35].

## 6. MicroRNAs

In the past 10 years, great importance has been given to the various functions of RNA, as not only it participates in the process of gene expression, but there are also different types of noncoding RNAs such as the so-called microRNAs (miRNAs) that play an important role in the regulation of gene expression in animals, plants, and viruses and have a crucial role in processes of cell differentiation, development, and proliferation, in cell death and in the acquisition and maintenance of a particular phenotype (e.g., tumor), among many other examples [36]. The first miRNAs were discovered through genetic research of the nematode* Caenorhabditis elegans*. This is why we intend to explain the role played by miRNAs, from their biogenesis to the control of the expression of some genes. These small RNAs associated with multienzyme complexes are used for the recognition of complementary sequences in target messenger RNA (mRNA). The functional interaction between both of them induces the degradation of mRNA and consequently translational repression, a mechanism considered as another form of epigenetic regulation [37].

## 7. Biogenesis of miRNAs

**Figure 1:**
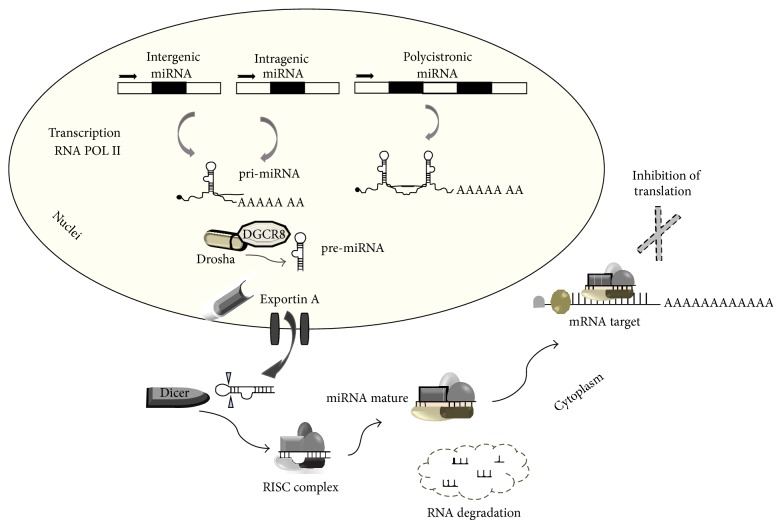
Biogenesis of miRNAs.

The biosynthetic pathway of miRNAs includes several stages: initially, miRNAs are transcribed by RNA polymerase II to generate precursor molecules called pri-miRNA, with a modification 5′ (7-methyl guanosine) and a tail of poly(A) at the 3′ end. These transcripts may be up to several kilobases in length. A single pri-miRNA may contain one or several miRNAs. These primary transcripts self-align to their sequence, forming stem-loop structures. Subsequently, these pri-miRNA are processed in the nucleus by a protein complex called “microprocessor,” formed by an RNase III called Drosha accompanied by the protein DiGeorge syndrome critical region gene 8 (DGCR8) (Figure 1), which recognizes the pri-miRNA and generates a smaller precursor known as pre-miRNA, of between 60 and 100 nucleotides in length, forming a stem-loop structure. Exportin-5 is a nuclear export protein that recognizes and transports pre-miRNAs to the cytoplasm. In the cytoplasm, the Dicer enzyme is involved; this is a second RNase III enzyme that separates the pre-miRNAs to generate the mature miRNA with a length of 18–24 nucleotides. RNA induces the activation of the RNA-induced silencing complex (RISC), in which the main component is the Argonaute protein, which includes a guide strand of miRNAs (Figure 1). The posttranscriptional silencing mediated by miRNAs occurs either due to the specificity of homologous mRNAs or when the guide miRNA joins the RISC complex and it in turn recognizes the target mRNA and represses gene expression through the imperfect (in animals and viruses) or perfect (in plants) coupling of the 3′ untranslated region UTR region (most of cases) of the target mRNA, preventing the production of the protein [36, 38].

It has been demonstrated that miRNAs have many biological functions. Their targets range from molecules involved in the signaling pathway of proteins, such as enzymes and transcription factors, to RNA-binding proteins. The diversity and abundance of target genes offer a number of possibilities and combinations and suggest that miRNAs and their targets form, as mentioned above, a complex regulatory network intertwined with other cellular networks such as the signal transduction, metabolic pathways, gene regulation, and protein interaction networks. Therefore, it is essential to understand the general principles of the regulation exerted by miRNAs to understand how they participate in the regulation of different cellular processes and, consequently, to understand their function at system level.

## 8. miRNAs as Molecular Markers

Since the discovery in the 90s of miRNAs as potent epigenetic regulators that have a general inhibitory effect on gene expression, they have opened a new era in the study of the regulation and development of cancer. This also started an exploration of the possible therapeutic applications of miRNAs. Although most miRNAs have not yet been characterized in terms of function and the signaling pathways regulated by them, certain mammalian miRNAs have emerged as critical regulators of stem cell function, self-renewal, epithelial-mesenchymal transition (EMT), initiation of cancer, resistance to therapy, and the promotion of metastasis [39].

## 9. Studies of miRNAs in Medulloblastomas

Studies of miRNAs and MB show that none of the miRNAs that have been studied are encoded on chromosome 17. Few of the target proteins of these miRNAs are from epidermal growth factor receptor (EGFR), B-cell lymphoma 2 (Bcl-2), and cyclin-dependent kinase 6 (CDK6) to solute carrier family 16, member A11 (SLC16A11); and others have not been validated. Several miRNAs have been associated with both poor and better prognoses when their levels are increased or decreased. Anyone can induce gain or loss of function. Some miRNAs are shown in Table 1 and summarize the information of four review articles published about miRNAs in MB [40–43].

## 10. Location of the Chromosome 17 miRNAs

Table 2 shows a review of the loci on chromosome 17 that, when mutated, may cause a disease. This illustrates a wide and diverse range of loci containing genes that may play an important role in the development of various diseases. This picture really changes when the loci of miRNAs encoded on chromosome 17 are located; these miRNAs can, through their multiple gene regulatory network, impact heavily on many diseases and disorders of the central nervous system development.

With this approach in mind, it would be possible to understand why the study of miRNAs can have a greater impact than the study of only the genes encoded on chromosome 17.

**Figure 2:**
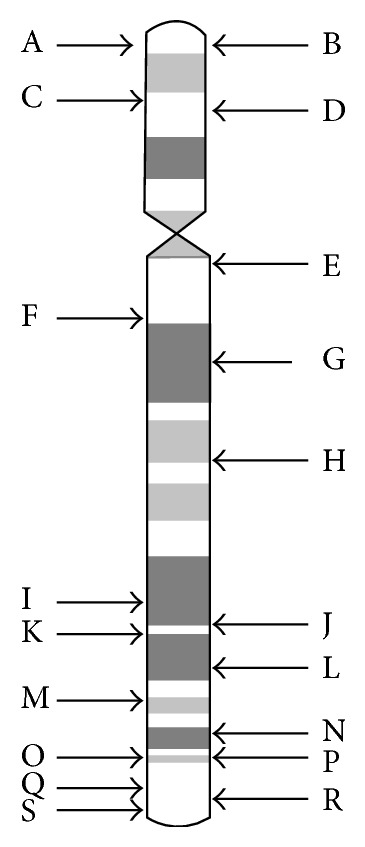
Location of the loci of the miRNAs described in Table 3, located on human chromosome 17.

Figure 2 shows the loci of the main miRNAs as unique genes encoded along chromosome 17, whose targets are, according to http://targetscan.org/, involved in the development and differentiation of the central nervous system (Table 3).

## 11. Clinical Significance of Some miRNAs

Our working group, based on the importance of chromosome 17 described above and on the proven fact that alterations or losses in genes of that chromosome in MB mark tumors with a poor prognosis, took on the task of choosing for study four miRNAs that we consider important for their potential involvement in MB. The expression alterations of these miRNAs have not been explored yet or their role in the different subtypes of MB and in the prognosis and survival of patients. We have a tissue bank of MB of Mexican pediatric patients, for whom there is no information regarding the expression of miRNAs in Mexico and Latin America; therefore, we consider it very important to conduct this study in next time and later integrate the information obtained into another article.

The potential clinical applications of these miRNAs should be evaluated based on their expression profile. If the interest is their utility in tumor tissues, it will be important to check their expression, comparing normal and tumor tissue. In the case of MB, it will be necessary to evaluate and check the expression between tumor and normal cerebellum samples. Hence, two possibilities arise: one is that the phenotype is overexpressed in the tumor compared to normal tissue. In this case, it means that this overexpression is causing a loss of function of some important proteins that is allowing/promoting tumor development. In this case, it would be appropriate to reduce the levels of the mature form of the miRNA to restore the function lost by the group of proteins whose mRNAs are targets for these miRNAs. In the contrary case, when the expression of the mature miRNA is reduced in the tumor tissue and is highly expressed in normal tissue, this strongly suggests that a function is gained by the expression of a group of proteins that allow the manifestation and maintenance of the tumor phenotype. In this case, it would appropriate to introduce the active form of the miRNA to again control the function and allow regulating the function of the tumor cells. Following this pattern, it is possible to compare pairs of deregulated microRNAs (overexpressed or underexpressed) between 2 different tumor phenotypes: primary tumor and metastasis; sensitive and drug-resistant phenotype, equal to radiotherapy; angiogenic and nonangiogenic; low and high tumor grade; adenocarcinoma and squamous cell carcinoma, and so forth.

### 11.1. miRNA-1253

The mature sequence of this miRNA is** agagaagaagaucagccugca**. An analysis of the target sequences of miRNA-1253 in http://targetscan.org/ reveals the presence of 330 transcripts and http://mirdb.org/ shows 466 conserved binding sequences of this microRNA. This analysis inevitably reveals a complex network of signals that are difficult to group. There is no report in the literature that describes an important role of this microRNA in any cellular process.

However, it is noteworthy that the group of mRNA targets contains the protein called neuroblastoma suppression of tumorigenicity 1 (NBL1). This protein is an antagonist of the differentiation factors bone morphogenetic factor 2 (BMP2) and BMP4 [44]. Factors BMP2 and BMP4 play an important role in MB; Iantosca et al. (1999), when evaluating the biological effects of these factors on DAOY cells (a medulloblastoma cell line), reported that exposure to BMP2 and BMP4 can reduce apoptosis and increase cell number. These responses are specific to these factors, as neither BMP3 nor transforming growth factor-beta 1 (TGF-*β*1) or glial cell derived neurotrophic factor (GDNF) is able to produce this effect. These results have an important potential clinical implication, as the increase of miR-1253 levels can induce a reduction in the concentrations of the NBL1 protein, which is an antagonist of the factors BMP2 and BMP4, thereby reducing apoptosis and increasing cell proliferation [45].

However, there is a contradictory report revealing that in retinoid-induced apoptosis in a model of tumor implant induced by a medulloblastoma cell line retinoids are able to induce the secretion of BMP2 in tumors of the cells sensitive to this agent, and that this signal is sufficient to produce apoptosis [46].

**Figure 3:**
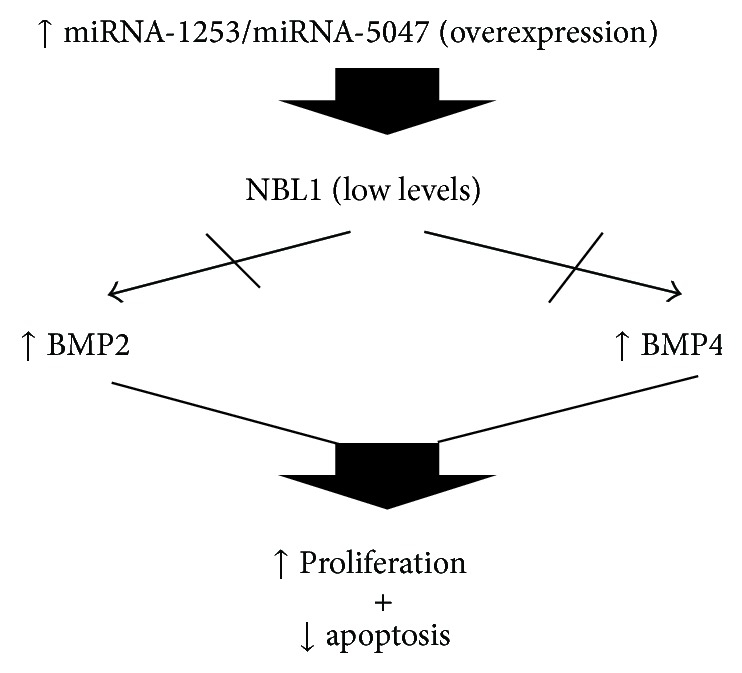
Pathway proposed in which miRNA-1253 and miRNA-5047 can deregulate some functions due to their overexpression and some cellular effects on proliferation and apoptosis in MB.

These results clearly reveal what the responses* in vitro* do not appear to correspond to the* in vivo* responses, so it is always necessary to validate and compare the results of the two assays. What is clear is that miRNA-1253 may have an important role in MB cells, negatively regulating an antagonist of the positive effects of the differentiation factors BMP2 and BMP4, thus causing a gain of function such as MB cell proliferation (Figure 3).

### 11.2. miRNA-2909

The mature sequence is** guuagggccaacaucucuugg**. This miRNA has around 97 miRNAs as targets according to http://targetscan.org/ and 151 according to http://mirdb.org/. A review of the literature reveals that the microRNA-2909 has been studied more extensively in cardiovascular disease, immune response, and the toxic response caused by arsenic, but no study has suggested a relation with central nervous system cells. However, it is important to mention that this microRNA is highly expressed in peripheral blood mononuclear cells isolated from patients with coronary artery disease and might be a diagnostic and prognosis marker for these patients with cardiovascular disease [47]. Moreover, Sharma et al. (2013) demonstrated that arsenic induces overexpression of miRNA-2909 and that this regulates the expression of important genes such as cyclin D1, reducing its levels and controlling the cell cycle [48].

Another effect of this microRNA is on the induction and maturation of T lymphocytes* in vitro* from mononuclear cells. MiRNA-2909 is capable of increasing the cell populations positive for cluster of differentiation 4 (CD4), CD25, and forkhead box P3 (Foxp3), as well as the populations of lymphocytes T helper 1 (Th1). Therefore, this miRNA can help in increasing the number of immune cells capable of protecting against viral infections and other pathogens [49].

The neuronal cell adhesion molecule (NrCAM) is among the target mRNAs of important proteins that have not yet been validated for this miRNA. It is a membrane protein widely expressed in the cerebellum, mainly during embryonic development. NrCAM is mainly expressed by Purkinje and Golgi cells in postnatal cerebellum, suggesting an important role in the maturation and stabilization of the synaptic connections in the cerebellum [50].

**Figure 4:**
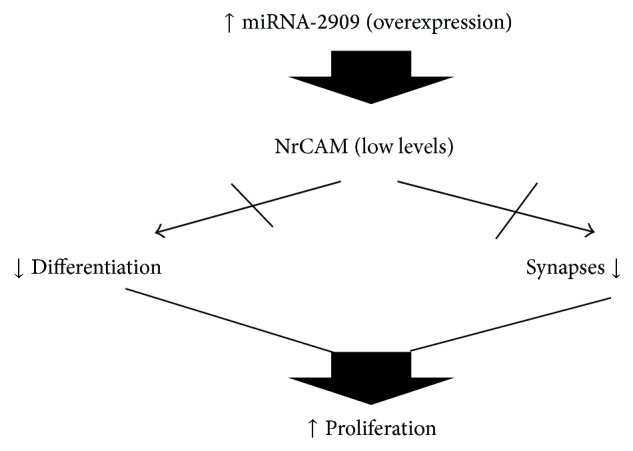
Pathway proposed in which overexpression of miRNA-1253 can affect NrCAM and results on proliferation in MB.

Thus, the overexpression of miRNA-2909 in MB can induce the degradation of this adhesion molecule and affect/activate some residual embryonic cells, which are the ones forming the MBs (Figure 4).

### 11.3. miRNA-5047

The mature sequence is** uugcagcugcgguuguaaggu**. There are no reports in the literature about this miRNA. A review of the mRNA sequences targeted by this microRNA revealed 356 transcripts according to http://targetscan.org/ and 333 according to http://mirdb.org/. It is noteworthy that this miRNA also targets the mRNA of the NBL1 protein, as in the case of miR-1253, so that an overexpression of these two miRNAs, miRNA-5047 and miRNA-1253, would ensure the degradation and significant reduction of the levels of this antagonist of the differentiation factors BMP2 and BMP4 and would have an effect on the apoptosis of MB cells (Figure 3). But one thing that stands out is that miR-5047 also targets Drosha ribonuclease III, which is involved in the processing of pri-microRNA into pre-miRNA in the nucleus. Thus, it is important to recognize that the expression of this microRNA can potentially affect the formation of pre-miRNAs and therefore the production of mature forms. The overall system for the processing and formation of miRNAs would be significantly affected.

### 11.4. miRNA-634

The mature sequence of this miRNA is** aaccagcaccccaacuuuggac**. An analysis of possible targets reveals 320 transcripts according to http://targetscan.org/ and 266 according to http://mirdb.org/. A review of the literature shows that this miRNA, and 39 others, is capable of controlling the human epidermal growth factor receptor 2 (HER2) signaling pathway and the cell replication pathway in breast cancer lines with amplification of this pathway [51].

Studies of the LN229 glioblastoma cell line are another example. Following the regulatory pathway of the cysteine-rich, angiogenic inducer 61 (CYR61) gene that activates cell proliferation and migration in these cells, with the aim of identifying a miRNA that controls it, it was found that miRNA-634 is among 3 miRNAs regulating this gene. This miRNA controlled cell proliferation but not migration. In addition, this miRNA can downregulate the mechanistic target of rapamycin (mTOR) pathway. These results demonstrate that miRNA-634 is an important regulator of the proliferation of glioblastoma cells [52]. It was also shown that this miRNA is overexpressed in chondrocytes of people without rheumatoid arthritis, so that a reduced or null expression of this microRNA may be involved in rheumatoid arthritis [53].

**Figure 5:**
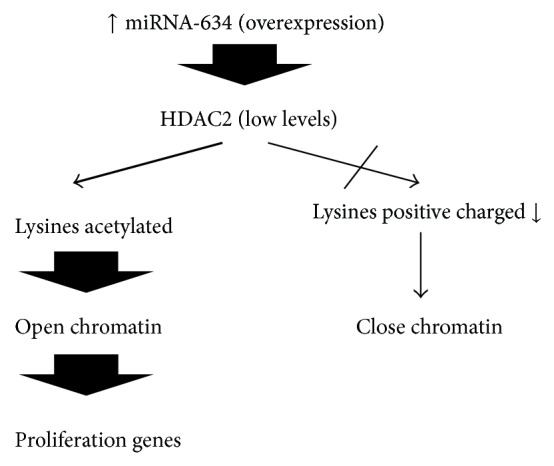
Pathway proposed in which overexpression of miRNA-634 can affect HDAC2 and open chromatin in MB.

Among the important target sequences of this miRNA that are implicated in MB, one of the most interesting gene is the mRNA of histone deacetylase 2 HDAC2. This gene encodes an enzyme that deacetylates histone lysines. This increased expression of HDAC2 might cause an increase of positively charged deacetylated lysines that may be related to transcriptionally closed chromatin, repressing some of the genes under the control of HDAC2 in MB (Figure 5). If levels of miRNA-634 increase, it reduces HDAC2 and it turns on open chromatin and transcription of genes of proliferation among others [54].

### 11.5. miRNA-636

The mature sequence of this miRNA is** ugugcuugcucgucccgcccgca**. The platform http://targetscan.org/ shows 656 sequences as potential targets for this miRNA, but http://mirdb.org/ shows only 173. There is a large discrepancy between the two platforms. When thinking about important targets in MB, it is worth noting that MB is thought to develop from cerebellar granule precursors. The SHH pathway is activated in these precursors and cell proliferation occurs with sustained activity of histone deacetylases HDACs. Several members of the HDAC family are expressed in the medulloblastoma, compared to what is observed in normal cerebellum. Thus, this miRNA may have important targets of upregulation through chromatin modification such as histone deacetylation [55].

**Figure 6:**
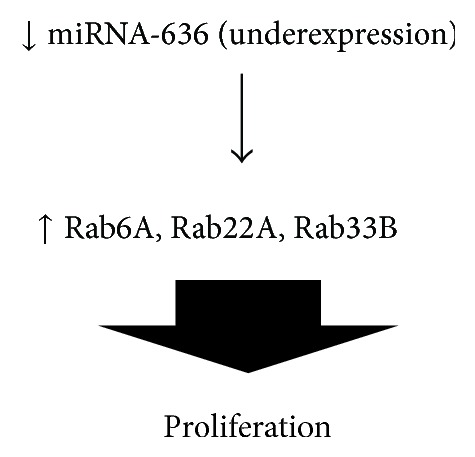
Pathway proposed in which underexpression of miRNA-636 can affect several Rab proteins and results on proliferation in MB.

A review of the main targets of miRNA-636 reveals important proteins of the rat sarcoma virus (Ras) pathway such as ras-related GTP binding proteins Rab6A, Rab22A, and Rab33B (Figure 6). This activation pathway may be involved in hepatic carcinoma. A low expression of miRNA-636 was observed in hepatocellular carcinoma cell samples, compared to normal liver samples. To verify this finding,* in vitro* experiments were performed with a liver carcinoma cell line, Hep3B; transfection with the mature form of miRNA-636 resulted in a significant reduction in cell proliferation and colony formation. This restoration of the levels of miRNA-636 significantly decreased the levels of Ras, supporting the results of the bioinformatic analysis of databases that predicts components of the Ras pathway as important targets of this miRNA [56].

## 12. Conclusions

After reviewing the historical progress of the description of molecular alterations in MB, based on the alterations of genetic pathways that have also been used to classify these tumors, it is important to note that, at present, the fact that Molecular Genetics is considered as a way of studying cellular functions from a different perspective from that of the pioneers of Classical Genetics and Cytogenetics, shows a clear picture of how Genetics itself has developed historically: from the guiding paradigm of genetic flow, one gene, and one protein, to the current paradigm of “one miRNA gene, multiple regulated genes.” Since the beginning of the sequencing of the human genome, only recently it was possible to clarify and understand the important functions of these noncoding RNAs that were known as “junk RNA.” If we make a clear and objective analysis, we must acknowledge that nature never produces unnecessary or “junk” actions or molecules because it has integrated millions of years of evolution that allowed it to know “intelligently” what it has and what it is good for; in addition, it will never work without profit, expending energy for something futile. The study of MB is only a pretext that helps understand more clearly how the new diagnostic, prognostic, and therapeutic approaches, based on Molecular Genetics, have started a revolutionary paradigm shift. What the study of Molecular Genetics makes clear is that the analysis based on miRNAs is teaching us how to integrate each element into a complex network such as that of biological systems, and that it is less and less possible to manipulate and explain things using the reductionist point of view with which we have learned to observe and study diseases such as cancer. miRNAs have shown increasingly solid therapeutic potential because they are not able to act on and modify the nucleus, as was thought in gene therapy. Thus, miRNAs fully respect the “Pandora box” of the nucleus, where the DNA resides and which must not be moved or altered in order to perform cleaner manipulations and obtain the desired effects. This is now possible thanks to miRNAs, which act in the cytoplasm and exert their regulatory action from there reducing the undesirable effects caused by inducing mutations by insertion or silencing by a reintroduced gene.

The potential clinical applications of miRNAs are focused in the following: if some miRNA encoded in chromosome 17 in MB is deregulated as overexpressed relative to cerebellum, the strategy will be to induce its degradation and control the loss of function as differentiation, apoptosis, and cell adhesion; it will be by transfecting with the antagonistic or antagomir sequence of the miRNA. If a miRNA is underexpressed and there is a gain of function in processes such as proliferation, migration, and metastasis, then an important strategy is to reintroduce the mature sequence to regain control of the function by negatively controlling it.

## Figures and Tables

**Table 1:** Summary of expression pattern of some miRNAs studied in MB and their potential clinical [40–43].

miRNA	Expression pattern	Potential clinical	Cellular targets	Cellular function
miRNA-let7g	UP	POOR	ND	ND

miRNA-9	DOWN	ND	Trkc	Increase apoptosis low proliferation

miRNA-10b	UP	ND	ND	ERBB2 overexpression

miRNA-17/92	UP	POOR	ND	SHH Pathway

miRNA-19a	UP	POOR	ND	Associated with high risk

miRNA-21	UP	ND	Pdcd4	Metastatic process

miRNA-23b	UP	BETTER	ND	WNT pathway

miRNA-25	UP	ND	P57	Tumor suppressive function

miRNA-30b/miRNA-30d	UP	ND	ND	8q24.22-q24.23 amplification

miRNA-31	DOWN	ND	ND	Associated with high risk

miRNA-34a	DOWN	ND	MAGE-A DII1 Notch1 Notch2	Increase apoptosis Increase cell cycle Decrease cell proliferation Increasing senescence

miRNA-96	DOWN/UP	ND/ND	AKT/ND	Cell cycle G0/G1, G2/ND

miRNA-100	UP	POOR	ND	Targets predicted in carcinogenesis

miRNA-106b	UP	POOR	ND	Anaplastic histology

miRNA-124a	DOWN	POOR	CDK6 SLC16A1	Cell cycle Glycolysis

miRNA-125a	DOWN	ND	Trkc	Increase apoptosis low proliferation

miRNA-125b	DOWN	POOR	Smo	SHH Pathway

miRNA-125p	DOWN	ND	Smo Gli1	SHH Pathway

miRNA-128a	DOWN/UP	BETTER/ND	Bmi-1/ND	Targeting oncogene Bmi-1/associated MYC

miRNA-128b	UP	ND	ND	Associated with MYC

miRNA-129	DOWN	ND	ND	ND

miRNA-135a/miRNA-135b	UP	ND	ND	ERBB2 overexpression

miRNA-148a	UP	BETTER	ND	WNT pathway

miRNA-153	DOWN	ND	ND	ERBB2 overexpression

miRNA-181b	UP	ND	ND	Associated MYC overexpression

miRNA-182	DOWN/UP	ND/ND	AKT/ND	Increase cell cycle G0 and G2, Decrease cell proliferation and migration/WNT pathway

miRNA-183~96~	DOWN/UP	ND/POOR	AKT/ND	Increase cell cycle G0 and G2, Decrease cell/ND proliferation and migration

miRNA-186	DOWN	ND	MYC	Decrease cell proliferation

miRNA-191	UP	POOR	ND	ND

miRNA-193a	UP	BETTER	ND	WNT pathway

miRNA-199b-5p	UP	BETTER	HES1	Notch signaling

miRNA-214	UP	POOR	SUFU	SHH pathway

miRNA-218	DOWN	POOR	EGFR Bcl-2	ND

miRNA-224/452	UP	POOR	ND	WNT pathway

miRNA-324-5p	DOWN	POOR	Gli1	SHH pathway

miRNA-326	DOWN	POOR	ND	ND

miRNA-365	UP	BETTER	ND	WNT pathway

miRNA-383	DOWN	ND	PRDX3	Increase apoptosis Increase cell cycle G1 Decrease cell proliferation

miRNA-512-2/miRNA-512-5p	DOWN	ND	MYC	Decrease cell proliferation

miRNA-548d-1/miRNA-548d-2	DOWN	ND	MYC	Decrease cell proliferation

miRNA-935	DOWN	ND	KIAA0232 SLC5A3 TBC1D9 ZFAND6	ND

ND, not determined.

**Table 2:** Loci of several genes on chromosome 17 and the main diseases induced when some of these genes suffer mutations.

LOCUS	Diseases
17p13.3	Retinitis pigmentosa
17p13.3	Platelet ADP receptor defect bleedings
Chr.17	Lambert-Eaton myasthenic syndrome
17p13	Type 2 diabetes mellitus
17p13.1	Congenital ichthyosiform erythroderma, Nonbullous
17p13.2	Miller-Dieker syndrome
17p13-p12	Liver failure
17p11.2	Van Buchem disease
17p11.2	Birt-Hogg-Dubé syndrome
17q11-q12	T cell immunodeficiency, alopecia, nail dystrophy
Chr.17	Endometrial stromal tumors
17q11.2	Alzheimer's disease
17q11.2	Acute promyelocytic leukemia
17q21-q22	White sponge nevus
17q21	Naxos syndrome
17q21	Narcolepsy
17q21	Sanfilippo syndrome type 2
17q21.32	Glanzmann's thrombasthenia
17q22	Early breast cancer
17q22-q23	Mulibrey nanism
17q22-q23	Meckel-Gruber syndrome
17q24-q25	Usher syndrome
17q25	Acute myeloid leukemia
17q25	Retinitis pigmentosa
17q25	Alveolar soft part sarcoma

**Table 3:** List of miRNAs encoded on chromosome 17 as single genes and their position, a target gene of each miRNA, its relevance for the nervous system, and its location on chromosome 17 illustrated in Figure 2.

mIRNA	Neurological importance	Location
**miRNA-3183**	Neurogenic differentiation 2 neuregulin 3	**A**

**miRNA-1253**	Neuroblastoma, suppression of tumorigenicity 1	**B**

**miRNA-4314**	Several neural targets	**C**

**miRNA-4521**	Neuropilin (NRP) and tolloid- (TLL-) like	**D**

**miRNA-4522**	Neurogenic differentiation 2	**E**

**miRNA-632**	Neurochondrin	**F**

**miRNA-2909**	Neuroblastoma RAS viral (v-ras) oncogene homolog	**G**

**miRNA-2117**	Neurofascin brain protein 44-like	**H**

**miRNA-3185**	Neural precursor cell expressed, developmentally down-regulated 4	**I**

**miRNA-4736**	Several neural targets	**J**

**miRNA-4729**	Several neural targets	**K**

**miRNA-4737**	Neurooncological ventral antigen 1	**T**

**miRNA-633**	Neurooncological ventral antigen 1	**M**

**miRNA-5047**	Neuroblastoma, suppression of tumorigenicity 1	**N**

**miRNA-634**	Neuroblastoma RAS viral (v-ras) oncogene homolog	**O**

**miRNA-636**	Neurooncological ventral antigen 1	**P**

**miRNA-4739**	Neurooncological ventral antigen 2	**Q**

**miRNA-657**	Neuroepithelial cell transforming 1	**R**

**miRNA-4525**	Neurochondrin	**S**

## Abbreviations

APC: Adenomatous polyposis coli gene
Bcl-2: B-cell lymphoma 2
BMP2: Bone morphogenetic factor 2
BMP4: Bone morphogenetic factor 4
CD4: Cluster of differentiation 4
CDK6: Cyclin-dependent kinase 6
CNS: Central nervous system
CYR6: Cysteine-rich, angiogenic inducer 61
DGCR8: DiGeorge syndrome critical region gene 8
EGFR: Epidermal growth factor receptor
EMT: Epithelial-mesenchymal transition
Foxp3: Forkhead box P3
GDNF: Glial cell derived neurotrophic factor
HDAC2: Histone deacetylase 2
HDACs: Histone deacetylases
HER2: Human epidermal growth factor receptor 2
MB: Medulloblastoma
mTOR: Mechanistic target of rapamycin
NBL1: Neuroblastoma suppression of tumorigenicity 1
NOTCH: Gene associated with notches
NRCAM: Neuronal cell adhesion molecule
PTCH: Patched gene
Ras: Rat sarcoma virus
RISC: RNA-induced silencing complex
SHH: Sonic hedgehog
SLC16A11: Solute carrier family 16, member A11
SMO: Smoothened
SUFU: Suppressor of fused
TGF-*β*1: Transforming growth factor-beta 1
Th1: Lymphocytes T helper 1
TP53: Tumor protein 53 gene
WNT: Wingless-type
UTR: Untranslated region.
